# Supplementary material for: SARS-CoV-2-neutralizing humoral IgA response occurs earlier but modest and diminishes faster compared to IgG response
Source: bioRxiv. 2022 Jun 9:2022.06.09.495422. Preprint. [Version 1] doi: 10.1101/2022.06.09.495422 (PMC9196114; doi:10.1101/2022.06.09.495422)
Supplement: 1 [file NIHPP2022.06.09.495422V1-supplement-1.pdf]

bioRxiv preprint doi: https://doi.org/10.1101/2022.06.09.495422; this version posted June 9, 2022. The copyright holder for this preprint (which was not certified by peer review) is the author/funder. This article is a US Government work. It is not subject to copyright under 17 USC 105 and is also made available for use under a CC0 license.

Supporting Information

S1 Fig. Four parameters curve fit model of the quantification of S1-binding antibody levels using the commercially available S1-binding IgA ELISA.

S2 Fig. High correlations of purified-IgG and -IgA neutralizing activities with S1-binding antibody levels.

The NT_{50} values against S1-binding IgG and IgA levels are shown in panels **A** and **C**, respectively, and nIgG-EC_{50} and nIgA-EC_{50} values against the S1-binding IgG and IgA are shown in panels **B** and **D**, respectively.

S3 Fig. Kinetics and the correlations of nasal SARS-CoV-2-S1-binding-IgA levels and total IgG and IgA amounts in serum.

The % SARS-CoV-2-S1-binding IgA levels in nasal swab samples were determined with the commercially available S1-binding IgA ELISA using a COVID-19-convalescent plasma's S1-binding IgA that was referred as 100%. (**A**) Temporal changes of the nasal S1-binding-IgA levels in over 18 days following the onset of the disease. (**B**) Correlation of % nasal S1-binding-IgA levels with that of sera/plasmas S1-binding IgA. Temporal changes of total human IgG and IgA levels following the diseases (**C** and **D**). Correlation of total human IgA levels with that of IgG is shown (**E**).

S4 Fig. COVID-19 mRNA-vaccine induces significant neutralizing activity and S1-binding antibody levels in COVID-19-experienced individuals.

The neutralizing activity of sera/plasmas, purified-IgG, and purified-IgA (**A**, **B**, and **D**, respectively) and the amounts of S1-binding IgG and S1-binding IgA (**C** and **E**, respectively) were compared between the pre- and post-vaccination.

bioRxiv preprint doi: https://doi.org/10.1101/2022.06.09.495422; this version posted June 9, 2022. The copyright holder for this preprint (which was not certified by peer review) is the author/funder. This article is a US Government work. It is not subject to copyright under 17 USC 105 and is also made available for use under a CC0 license.

S5 Fig. Correlations of sera/plasmas, purified-IgG, and -IgA neutralizing activities with S1-binding antibody levels.

The NT_{50} values against (**A**) nIgG-EC_{50} values, (**B**) nIgA-EC_{50} values, (**D**) S1-binding-IgG level (S1-binding IgG), and (**F**) S1-binding-IgA level are plotted. Note that neutralizing activity of IgG primarily contributes to sera/plasmas SARS-CoV-2-neutralizing activity compared to that of IgA (**A**, **B**, and **C**) in previously-COVID-19-contracted individuals following COVID-19 mRNA vaccination.

bioRxiv preprint doi: https://doi.org/10.1101/2022.06.09.495422; this version posted June 9, 2022. The copyright holder for this preprint (which was not certified by peer review) is the author/funder. This article is a US Government work. It is not subject to copyright under 17 USC 105 and is also made available for use under a CC0 license.

S1 Table. Experimental therapeutic agents used in the COVID-19 group.

	All patients (n = 14)	Moderate (n = 7)	Severe (n = 7)
Experimental therapeutic agents			
Remdesivir (RDV)	4 (28.6%)	2 (28.6%)	2 (28.6%)
Lopinavir/ritonavir (LPV/r)	2 (14.3%)	1 (14.3%)	1 (14.3%)
Hydroxychloroquine (HCQ)	3 (21.4%)	2 (28.6%)	1 (14.3%)
HCQ + Azithromycin (AZM)	3 (21.4%)	0 (0%)	3 (42.9%)
inhaled Ciclesonide (CIC)	1 (7.1%)	1 (14.3%)	0 (0%)
Favipiravir (FPV)	1 (7.1%)	0 (0%)	1 (14.3%)
None	2 (14.3%)	1 (14.3%)	1 (14.3%)
Corticosteroid use			
Hydrocortisone (HDC)	4 (28.6%)	0 (0%)	4 (57.1%)
Methylprednisolone (mPSL)	1 (7.1%)	0 (0%)	1 (14.3%)
PMX-DHP	3 (21.4%)	0 (0%)	3 (42.9%)

Abbreviation: PMX-DHP; polymyxin B-immobilized fiber column direct hemoperfusion
